# The Utility of Endoscopic Biopsies in Patients with Normal Upper Endoscopy

**DOI:** 10.1155/2016/3026563

**Published:** 2016-07-10

**Authors:** Anouar Teriaky, Abdullah AlNasser, Carolyn McLean, James Gregor, Brian Yan

**Affiliations:** ^1^Department of Medicine, Division of Gastroenterology, Western University and London Health Sciences Centre, London, ON, Canada N6A 5W9; ^2^Department of Pathology, Western University and London Health Sciences Centre, London, ON, Canada N6A 5W9

## Abstract

*Background and Aims*. Upper endoscopy is a valuable tool in the workup of gastrointestinal (GI) complaints. The purpose of this study is to determine cost and yield of taking biopsies in a normal upper GI tract.* Methods*. This is a retrospective study where all upper GI biopsies were identified between May 2012 and April 2013, at a tertiary care center. Clinical, procedural, and pathology reports were reviewed to identify patient demographics, procedure information, and pathology diagnosis.* Results*. Biopsies of the upper GI tract were taken in 1297 patients with normal upper endoscopies. In patients with normal upper endoscopy, 22% of esophageal, 44% of gastric, and 12% of duodenal biopsies were abnormal. The most frequent abnormality was reflux esophagitis in 16% of esophageal biopsies, chronic gastritis in 23% of gastric biopsies, and increased intraepithelial lymphocytes in 6% of duodenal biopsies. The additional cost for taking biopsies in a normal upper GI tract for a diagnosis of eosinophilic esophagitis was $2963 Canadian (CAD),* H. pylori* associated gastritis was $1404 CAD, and celiac disease was $3024 CAD.* Conclusions*. The yield of biopsy in normal upper endoscopy varied with location, but the additional expense can be costly and should be tailored to appropriate clinical situations.

## 1. Introduction

Upper gastrointestinal (GI) disorders are highly prevalent in the general population and cause significant morbidity [1–3]. The disease burden leads to impairment in quality of life and considerable healthcare costs [4–6]. GI symptomatology tends to be nonspecific and poorly correlates with organic etiology seen on endoscopy [7]. The workup in determining the etiology of upper GI complaints includes laboratory investigations and various imaging modalities. Endoscopy is not always required as a first line investigation except when alarm symptoms are present or the patient's age is greater than 50 years [8].

About 2% of the adult population in the United States (US) requires upper endoscopy (EGD) each year [9]. Endoscopy remains an essential diagnostic tool providing a more in depth visual assessment of GI mucosa than any other modality and allows for the sampling of tissue, which can be further assessed by a pathologist. While abnormal endoscopic appearance may indicate a disease state, biopsy will ultimately determine if this is the case. In cases where the GI mucosa appears visually normal with endoscopy, the use of biopsy may still be beneficial in determining microscopic disease [10–12]. The value of taking biopsies in a normal GI tract can be both costly and time consuming, and the yield of biopsies in an endoscopically normal upper GI tract varies depending on the indication for the procedure. Complications from taking biopsies tend to be minimal.

In the lower GI tract, obtaining biopsies from an endoscopically normal terminal ileum during colonoscopy adds little additional information [13–15]. However, colonic biopsies of an endoscopically normal colon yield important information on etiology in patients presenting with chronic diarrhea [16–20]. The cost of healthcare continues to escalate with a significant proportion of gastroenterology related healthcare costs attributed to endoscopy [21]. In the US the annual cost of EGDs is approximately 12.3 billion US dollars [9]. The additional institutional cost of biopsies and pathological assessment increases the cost substantially, yet it is unknown whether this cost is worthwhile as the pathological findings often do not alter clinical management.

A substantial number of upper GI tracts have biopsies taken during EGD even when the mucosa appears macroscopically normal. The augmented yield of taking biopsies in a normal upper GI tract and the associated cost has not been well studied. The purpose of this study is to determine the yield of taking biopsies in a normal upper GI tract in patients requiring EGD and the incremental cost per significant upper GI disease identified.

## 2. Methods

This is a retrospective study performed at London Health Sciences Centre (LHSC) and St. Joseph's Health Care (SJHC) in London, Ontario, Canada. Both institutions are academic tertiary referral centers for gastroenterology. Inclusion criteria included all patients with EGDs performed with biopsies taken between May 1, 2012, and April 30, 2013. Exclusion criteria included patients with missing clinical, procedural, or pathology information and nonduodenal small bowel biopsies. If a patient had more than one EGD with biopsies performed in the study period, only the first EGD was included. All biopsies of the upper GI tract were identified from a pathology database. Upper GI tract biopsies were categorized into esophageal, gastric, or duodenal. Using patient identifiers, a manual review of all patient clinical, procedural, and pathology reports was completed to extrapolate patient demographics, procedural information, and pathology diagnoses.

Patient demographics consisted of age, sex, nonsteroidal anti-inflammatory drug (NSAID) or aspirin (ASA) use, anticoagulant use, and proton pump inhibitor (PPI) or H2 receptor antagonist (H2RA) use. Anticoagulants identified were warfarin, clopidogrel, and the novel oral anticoagulants. Procedural information identified included procedure indication and endoscopic findings in the esophagus, stomach, and duodenum. An EGD was considered abnormal if any macroscopic luminal findings were identified including mild erythema. Only one indication was identified per patient. A hiatus hernia was not considered a clinically significant abnormality. The number of biopsies taken at each site was unavailable and could vary from one to several. Pathology reports consisted of any histological abnormalities identified with a final pathologic diagnosis. The majority of EGDs were performed by gastroenterologists while a small proportion were performed by surgeons. Several specialized GI pathologists analyzed pathology reports. Pathology residents and gastroenterology fellows participated in a proportion of cases under the supervision of a staff physician.

Ontario Ministry of Health billing codes and institutional fees were used to determine the cost of biopsies [22]. The gastroenterology biopsy billing code was $15.10 Canadian (CAD). The pathology billing code per site biopsied consisted of a technical component of $16.54 CAD and a professional component of $48.65 CAD. The institutional fee consisted of the cost of the biopsy forceps of $11.50 CAD and the formalin bottle of $0.56 CAD. If more than one site was biopsied during an EGD, overall cost would drop as some components of cost only needed to be included once in the calculation. This was factored into the final calculation of cost to prevent overestimation. The mean cost per esophageal biopsy was $78.22 CAD, gastric biopsy was $80.34 CAD, and duodenal biopsy was $79.83 CAD. The cost of the endoscopic procedure was not factored into cost of taking biopsies. We determined the cost per positive finding on histology by using the yield of abnormal findings in order to determine the amount of normal biopsies required to get an abnormal finding and determining cost based on this amount.

The yield of abnormal histology on normal upper endoscopy was identified by sites biopsied or diagnosis and expressed as a percentage and cost per positive finding. Macroscopically normal and abnormal EGDs were separated in order to determine clinical predictors of normal EGD. Variables included in the multivariable analysis were age <50 years, sex, NSAIDS or ASA, anticoagulation, PPI, H2RA, and EGD indication. These same variables were used when determining clinical predictors of abnormal histology for macroscopically normal EGDs. Data was collected in excel spreadsheets. Statistical analysis was performed with multivariable logistic regression presented as odds ratio with 95% confidence intervals. SAS 9.4 was used for statistical analysis and a *P* value < 0.05 was considered significant.

## 3. Results

**Figure 1:**
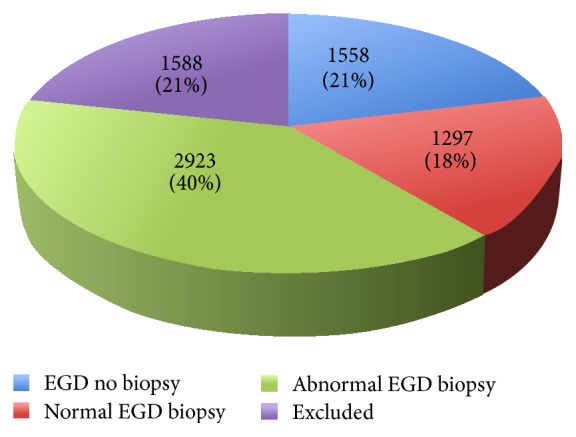
All EGDs performed at LHSC and SJHC between May 2012 and April 2013. Excluded EGDs included patients that had more than one EGD within the year or missing clinical, procedural, or pathological information.

A total of 7366 EGDs were performed between May 2012 and April 2013 at LHSC and SJHC. There were 5808 EGDs (79%) with biopsies performed. Of these EGDs, 1297 were macroscopically normal, 2923 were macroscopically abnormal, and 1588 were excluded from this study (Figure 1). The 1588 EGDs excluded from the study were due to repeated procedures within the same year, nonduodenal small bowel biopsies, or insufficient clinical, procedural, or histologic information. Patient demographics and indication for EGD for patients with macroscopically normal and abnormal EGDs can be seen in Table 1. A multivariable logistic regression of the clinical predictors of macroscopically normal EGDs is listed in Table 2.

The yield of any abnormal histology when taking biopsies in an endoscopically normal upper GI tract was 22% for the esophagus, 44% for the stomach, and 12% for the duodenum.  Table 3 lists the yield for GI diagnosis identified through taking biopsies in an endoscopically normal upper GI tract and the additional cost incurred to make each diagnosis.

A multivariable logistic regression identifying the clinical predictors of abnormal biopsy on macroscopically normal EGDs can be seen in Table 4. PPI therapy did not provide protection against abnormal histology in the esophagus or stomach in normal endoscopy on multivariable analysis. The yield of an abnormal pathologic diagnosis and cost per positive finding in cases of macroscopically normal EGDs based on the clinical indication can be seen in Table 5. In patients with normal EGDs with increased intraepithelial lymphocytes on duodenal biopsy there was a positive tissue transglutaminase antibody in only 10% of the patients tested, which could indicate latent celiac disease. In patients with normal EGDs with a duodenal biopsy consistent with celiac disease the tissue transglutaminase antibody was positive in 95% of the cases tested. Of the patients that underwent EGDs for anemia, 60% of patients did not have laboratory investigations consistent with iron deficiency anemia when the data was available.

### 3.1. Cost of Biopsying a Normal Upper GI Tract

The additional cost incurred when taking biopsies of an endoscopically normal upper GI tract per abnormal histologic diagnosis was $356 CAD for the esophagus, $183 CAD for the stomach, and $665 CAD for the duodenum (Table 3). For 1297 normal EGDs, 2474 biopsies were taken and our institution incurred an estimated $200,000 annual cost not including the cost of the endoscopic procedure. Taking biopsies in an endoscopically normal upper GI tract did not change clinical management in 94% of cases.

## 4. Discussion

This study evaluated the utility of taking biopsies in the upper GI tract during a macroscopically normal EGD to determine the incremental increase in yield and cost. The literature has been lacking in assessing this question when it comes to the upper GI tract. With the need for economic constraints, cutting back on unnecessary costs in clinical practice has become essential [9]. Pathology departments are also already inundated with time constraints from their busy workload. Therefore, determining if biopsies are necessary when endoscopy is normal is important. Parameters of macroscopically normal EGD included younger age, female sex, lack of NSAID or anticoagulation, and nonspecific GI indications for EGD. This is consistent with prior studies identifying older age, the use of NSAIDS or anticoagulation, and alarm features as predictors of abnormal EGDs except that anemia was a predictor of normal EGD in this study [8, 23–26].

Predictors of abnormal histology on normal upper endoscopy were dependent on the site of biopsy. The lack of NSAID use predicted abnormal esophageal histology. Taha et al. interestingly showed that long term NSAID use led to less histological abnormalities in the esophagus [27]. Predictors of abnormal gastric biopsy included older age and anticoagulation use. Aging has been shown to lead to increased abnormalities on gastric biopsy [28]. Predictors of abnormal duodenal biopsy were older age while endoscopic indications of anemia and dyspepsia make it less likely to have abnormal histology. Dyspepsia is a nonspecific complaint more likely to yield a normal biopsy than not. However, other studies have shown that anemia is more likely to yield an abnormal duodenal biopsy especially when celiac disease is suspected [29]. The likely explanation of anemia being a predictor of a normal EGD and normal duodenal biopsy in this study is that 60% of patients with laboratory investigations available did not have evidence of iron deficiency anemia when the data was available in a subset of patients. Iron deficiency anemia has been shown to be a clinical predictor of abnormal EGD and duodenal histology. Unfortunately, we did not have iron studies on all patients with anemia to statistically assess if the population with iron deficiency was more likely to have an abnormal EGD and duodenal biopsy in our study. The prevalence of a GI cause of anemia in patients without iron deficiency anemia is significantly lower than patients with iron deficiency anemia [30]. However, patients without iron deficiency anemia may still require endoscopy if there is evidence of acute or subacute GI bleeding where there is insufficient time to deplete iron stores.

The yield of abnormal histology when taking biopsies in an endoscopically normal upper GI tract varied with site being highest in the stomach at 44% and lowest in the duodenum at 12%. Our institute spent approximately $200,000 CAD to biopsy endoscopically normal upper GI tracts in 1297 patients with 94% of biopsies not changing management. The cost associated per positive diagnosis was substantial in some cases costing thousands of dollars. The yield and cost appeared to improve in cases where there was a targeted indication. For example, patients with a clinical indication of dysphagia had an increased yield of eosinophilic esophagitis and patients with diarrhea had an increased yield of celiac disease. To diagnose one case of celiac disease in patients presenting with dyspepsia, the cost was near $4000, and in anemia near $8000, but in those with diarrhea, the cost was at a more reasonable $1300. While these costs are less than the cost to investigate patients with chest pain or screen for malignancy, the clinical significance of taking biopsies in a normal upper GI tract is much less meaningful than these other necessary investigations especially when there is not a targeted indication [31–34]. The costs presented in this study are lower than other studies assessing the economic impact of current endoscopic practice [21, 35]. The cost was based on Ministry of Health billing codes in Ontario and institutional costs. This cost is not accurately representative of academic institutions where pathologists are salaried. This cost is also an underestimate, as some variables could not be reliably translated into the final cost. Further, 21% of EGDs were excluded for various reasons resulting in an underestimation of cost.

While we do not support avoiding taking biopsies in all macroscopically normal upper GI tracts, we believe that a tailored indication that alters management is the best indication. This is consistent with the recently released American Gastroenterological Association (AGA) guidelines on taking biopsies from a normal upper GI tract for an indication of dyspepsia [36]. The AGA guidelines recommended against taking esophageal biopsies in this case but recommended taking gastric biopsies only if the* H. pylori* status is unknown and duodenal biopsies in the absence of other symptoms of celiac disease only in immunocompromised patients.* H. pylori* should be tested with serology and treated if positive while patients with celiac disease especially those at low risk should have serology first before endoscopy to assess for these diseases.

Intraepithelial lymphocytosis in the duodenum is a nonspecific finding with a diverse differential diagnosis including early celiac disease [37]. In this study, of the patients with intraepithelial lymphocytosis, 90% did not have a positive tissue transglutaminase. The 10% that did may have had latent celiac disease. This is consistent with prior studies assessing the yield of celiac disease in patients with only intraepithelial lymphocytosis [38]. Even in this case, the yield of patients with celiac disease would only increase by 0.6%. Of the patients that did have a biopsy consistent with celiac disease, 95% had a positive tissue transglutaminase antibody, which is consistent with the specificity of the test and confirms the important role in screening this antibody plays [39]. Gastric intestinal metaplasia was found in a small proportion of patients with macroscopically normal EGDs. While there is some evidence suggesting a small risk of further progression to dysplasia and cancer, further studies are required to determine appropriate surveillance intervals once gastric intestinal metaplasia is identified [40].

Limitations of this study include its retrospective nature, which can lead to bias, confounding, and an inability to retrieve some results. This study is also a single center experience. As a wide variety of endoscopists performed the EGDs, variations may exist in the interpretation of normal, which can lead to skewing the outcomes. Several GI pathologists also participated in this study and can also alter the results by variations in interpretation of the histology. Further to this, the amount of biopsies taken at each site was variable and in patchy diseases taking less biopsies may lead to underdiagnosis. However, even with the limitations of this study, there are still important deductions that can be made.

In conclusion, predictors of normal upper endoscopy included younger age, female sex, and nonspecific GI procedure indications while predictors of abnormal histology varied with site. The yield of the biopsy in normal upper endoscopy varied with location. Yield and subsequent cost benefit improves with targeted indications. Practitioners need to be aware of the additional expense incurred by biopsies of normal upper GI tract and should tailor biopsies to appropriate situations that alter clinical practice, such as dysphagia for eosinophilic esophagitis,* H. pylori* for dyspepsia when serology status is unknown, or diarrhea for celiac disease.

## Figures and Tables

**Table 1:** Patient demographics and indication for EGD for patients with macroscopically normal and abnormal EGDs.

Demographic (mean, percentage, or number)	Normal EGD [*N* = 1297]	Abnormal EGD [*N* = 2923]
Age (years)	44	57

Sex (%)	Male: 32%	Male: 49%
	Female: 68%	Female: 51%

ASA/NSAID (%)	14%	26%

Anticoagulation (%)	3%	7%

PPI (%)	47%	49%

H2RA (%)	2%	2%

*EGD indication*		
Dyspepsia	554 (43%)	721 (25%)
Heartburn	164 (13%)	344 (12%)
Anemia	163 (12%)	336 (11%)
Diarrhea	91 (7%)	88 (3%)
Nausea/vomiting	88 (7%)	136 (5%)
Dysphagia	65 (5%)	368 (12%)
GI bleed	35 (3%)	264 (9%)
Chest pain	20 (2%)	27 (1%)
Weight loss	17 (1%)	44 (2%)
Other	100 (8%)	595 (20%)

EGD = upper endoscopy, ASA = aspirin, NSAID = nonsteroidal anti-inflammatory drug, PPI = proton pump inhibitor, H2RA = H2 receptor antagonist, and GI = gastrointestinal.

Other indications: odynophagia, halitosis, hiccups, globus, screening or surveillance for malignancy, radiologic abnormalities, food bolus, chronic cough, or research study.

**Table 2:** Clinical predictors of macroscopically normal EGDs on multivariable logistic regression.

Variable	Odds ratio	95% confidence interval	*P* value
Age <50 (years)	2.3	2.0–2.7	<0.0001
Female	1.7	1.4–1.9	<0.0001
No NSAID	1.6	1.3–1.9	<0.0001
No anticoagulation	1.8	1.2–2.6	0.0029
Anemia	3.1	2.4–4.1	<0.0001
Diarrhea	3.9	2.7–5.5	<0.0001
Dyspepsia	2.9	2.3–3.6	<0.0001
Heartburn	2.0	1.5–2.6	<0.0001
Nausea and vomiting	2.4	1.7–3.4	<0.0001

EGD = upper endoscopy, NSAID = nonsteroidal anti-inflammatory drug.

**Table 3:** Pathological diagnosis on biopsy of normal upper GI tract and cost per positive finding.

Pathological finding	Abnormal histology (%) (absolute number)	Cost/positive finding ($ CAD)
*Esophagus*		
Reflux esophagitis	16% (81/502)	$489
Eosinophilic esophagitis	3% (13/502)	$2963
Barrett's esophagus	1% (4/502)	$8889

*Stomach*		
Chronic gastritis	23% (243/1054)	$351
Chronic gastritis with IM	4% (42/1054)	$2282
HP gastritis	6% (62/1054)	$1404
HP gastritis with IM	1% (12/1054)	$6086
Reactive gastropathy	7% (69/1054)	$1217
Reactive changes	1% (15/1054)	$6086

*Duodenum*		
Increased IEL	6% (54/918)	$450
Duodenitis	2% (22/918)	$3501
Celiac disease	3% (25/918)	$3024
Lymphoid hyperplasia	0.5% (5/918)	$16631

IM = intestinal metaplasia, HP = *Helicobacter pylori*, and IEL = intraepithelial lymphocytes.

The percentages expressed in this table identify the yield of the diagnosis in patients with macroscopically normal EGDs that were biopsied.

**Table 4:** Clinical predictors of abnormal biopsy on macroscopically normal EGDs on multivariable logistic regression.

Variable	Odds ratio	95% confidence interval	*P* value
*Esophageal biopsy*			
No NSAID	3.8	1.1–12.6	0.03

*Gastric biopsy*			
Age <50 (years)	0.6	0.4–0.7	<0.0001
No anticoagulation	0.4	0.2–0.9	0.03

*Duodenal biopsy*			
Age <50 (years)	1.6	1.0–2.6	0.04
Anemia	0.4	0.2–0.9	0.03
Dyspepsia	0.3	0.2–0.6	0.001

NSAID = nonsteroidal anti-inflammatory drug, EGD = upper endoscopy.

**Table 5:** Yield of pathologic abnormality and cost per positive finding on macroscopically normal EGDs based on the clinical indication.

Indication	Pathologic finding	Histologic abnormality (%) (absolute number)	Cost/positive finding ($ CAD)
Dysphagia	Reflux esophagitis	25% (14/56)	$313
	Eosinophilic esophagitis	11% (6/56)	$711

Heartburn	Reflux esophagitis	21% (24/113)	$372
	Eosinophilic esophagitis	0% (0/113)	—

Dyspepsia	Reflux esophagitis	15% (33/220)	$521
	Chronic gastritis	27% (140/520)	$298
	HP gastritis	6% (31/520)	$1404
	Celiac disease	2% (8/420)	$3992

Anemia	Reflux esophagitis	0% (0/7)	—
	Chronic gastritis	30% (29/97)	$268
	HP gastritis	15% (15/97)	$536
	Duodenitis	1% (2/151)	$7983
	Increased IEL	7% (11/151)	$1140
	Celiac disease	1% (2/151)	$7983

Diarrhea	Increased IEL	7% (6/91)	$1140
	Celiac disease	6% (5/91)	$1331

Nausea and vomiting	Reflux esophagitis	7% (3/41)	$1117
	Eosinophilic esophagitis	2% (1/41)	$3911
	Chronic gastritis	24% (20/85)	$335
	HP gastritis	6% (5/85)	$1404

GI bleed	Reflux esophagitis	0% (0/2)	—
	Chronic gastritis	27% (8/30)	$298
	HP gastritis	10% (3/30)	$803
	Duodenitis	5% (1/21)	$1597

The percentages in this table indicate the yield of an abnormality found on a macroscopically normal EGD with a targeted indication.
